# Harnessing secondary metabolites of endophytic microbes: a next-generation biopesticide for crop disease management

**DOI:** 10.3389/fmicb.2025.1705702

**Published:** 2026-01-22

**Authors:** Gulafsha Parveen, Waquar Akhter Ansari, Navin Kumar, Durgesh Kumar Jaiswal

**Affiliations:** 1 Department of Biotechnology, Graphic Era (Deemed to be University), Dehradun, India; 2 Marwadi University Research Centre, Marwadi University, Rajkot, Gujarat, India; 3 Department of Biosciences, Graphic Era (Deemed to be University), Dehradun, India

**Keywords:** endophytic microbes, secondary metabolites, biopesticides, plant disease management, sustainable agriculture, plant–microbe interactions

## Abstract

This review highlights the potential of endophytic microorganisms and their secondary metabolites as innovative biopesticides for sustainable disease management in agriculture. Agriculture faces substantial challenges from phytopathogens, resulting in significant economic losses worldwide, which are typically addressed with synthetic pesticides that pose environmental and health hazards. Endophytic microorganisms residing within plant tissues without inducing disease provide a natural defence alternative by synthesising a variety of beneficial secondary metabolites, including alkaloids, terpenoids, phenolics, and peptides. These chemicals serve as ecological mediators, directly inhibiting pathogens, promoting plant systemic resistance, and improving nutrient absorption and stress resilience. The review elucidates the biosynthesis routes of these metabolites, their ecological functions, and the symbiotic chemical interactions between endophytes and host plants that enhance plant growth and defence. Bacterial endophytes, including *Bacillus* and *Pseudomonas*, generate lipopeptides that compromise pathogen membranes and to improve plant immunity, whereas fungal endophytes, such as *Trichoderma* and *Penicillium*, produce antifungal and insecticidal agents. The manuscript additionally examines the molecular mechanisms that govern these relationships, encompassing phytohormonal signalling and quorum sensing. While the potential of endophytic microorganisms as biopesticides is promising, significant gaps remain in our understanding of their long-term ecosystem effects, molecular mechanisms, and scalable manufacturing techniques. This review highlights the importance of comprehensive research to fully harness the biotechnological potential of endophytes. Integrating their secondary metabolites into crop protection strategies could reduce our reliance on chemical pesticides, promoting environmental sustainability and food security. Understanding the long-term ecosystem effects of endophytic microorganisms is crucial for bolstering resilient agricultural systems globally.

## Graphical abstract

**Figure:**
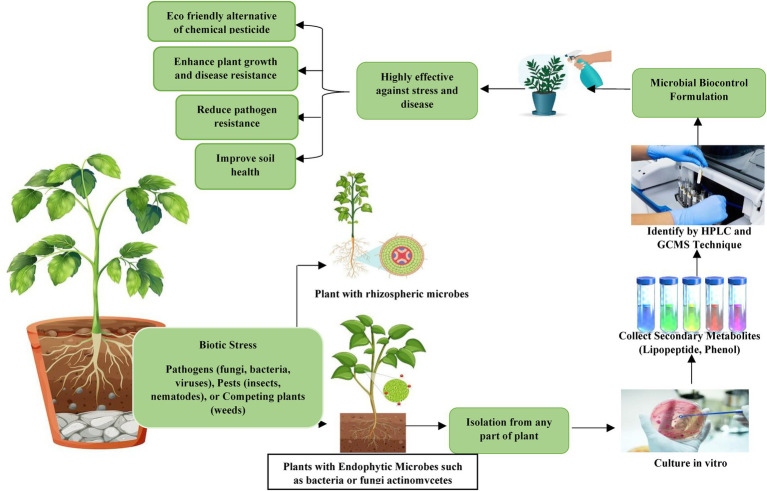

## Highlights

- Endophytic microbes, with their potential to serve as biocontrol tools against a variety of plant diseases, underscore the urgency and importance of our research in this field.
- Secondary metabolites that endophytic fungi produce are essential for their antifungal properties and defence mechanisms against phytopathogens.
- Endophytic microbes secrete secondary metabolites such as lytic enzymes, lipopeptides, flavonoids, and antibiotics that can prevent the growth of pathogens.
- Numerous methods for exploring endophytic microbes and plant interactions are essential for defining their mechanisms and enhancing their application in agriculture.

## 1 Introduction

Agriculture is fundamental to global sustenance and economic stability, particularly in nations like India, where it supports 52% of the population and contributes 20% to the nation’s GDP (Kalamkar, 2009; Smolińska, 2019). However, agricultural productivity is continually challenged by phytopathogens, including bacteria, viruses, fungi, and plant-parasitic nematodes, resulting in annual economic losses of billions of dollars. Global estimates attribute approximately 25% of agricultural losses to these biotic stressors (Junaid and Gokce, 2024), significantly impacting food availability and the financial viability of farming communities (Al-Sadi, 2021; Islam, 2025). For instance, *Fusarium graminearum* incites *Fusarium* head blight (FHB) in *Triticum aestivum* (Viviani et al., 2025), Tomato mosaic virus compromises tomato yields (Kobayashi et al., 2024), *Magnaporthe oryzae* causes devastating rice blast (Moin et al., 2024), and *Meloidogyne incognita* induces root-knot disease across various crops (Khan et al., 2024). Conventional disease management relies on synthetic pesticides such as organophosphates, carbamates, and pyrethroids; however, their overuse poses environmental and health risks, necessitating the exploration of sustainable alternatives (Le Cocq et al., 2017).

Within their natural ecosystems, plants interact with a diverse range of organisms, including microorganisms, that profoundly influence their survival and overall fitness (Santoyo et al., 2016). Among these, endophytic microorganisms residing within plant tissues without causing overt disease symptoms have emerged as a beacon of hope in the battle against phytopathogens. These endophytic communities, encompassing fungi, archaea (Fadiji et al., 2020), bacteria (Hardoim et al., 2015), and oomycetes (Larousse and Galiana, 2017), establish a symbiotic continuum that influences plant growth, nutrient uptake, and defence against pathogens. The intimate association between endophytes and their host plants facilitates intricate chemical communication, mediated by the production of specialised metabolites (Aly et al., 2011), which act as signalling molecules that govern communication, competition, and defence mechanisms (Ola et al., 2013). One of the most appealing approach to developing sustainable agriculture is the use of plant growth promoting bacteria to enhance the soil and plant health, and agricultural productivity. Actinomycetes are bacteria that are well known for their use in wheat, rice, beans, chickpeas, and peas. They also produce secondary metabolites of economic relevance and play a significant part in PGP and plant protection (Swarnalakshmi et al., 2016). Co-culturing studies of bacterial-fungal interactions have demonstrated the potential to generate diverse secondary metabolites (Abdalla et al., 2017), which play roles in biocontrol, disease suppression, and stress tolerance (Firdous et al., 2019). Biological control is being explored as a supplemental or alternative method of lowering the use of chemicals in agriculture (Liu et al., 2010). Due to their ecological niche, endophytes may have a more direct and consistent beneficial effect on plants than soil microorganisms (Gao et al., 2021) because endophytic microbes are not exposed to fluctuating external environmental conditions, such as temperature, soil pH, and water (Ameen et al., 2024).

These metabolites, broadly classified as terpenoids, phenolics, alkaloids, polyketides, and non-ribosomal peptides, exhibit a range of activities that impact ecological competition and symbiosis (Jayaprakashvel and Mathivanan, 2011). Compared to soil microorganisms, endophytes may exert a more pronounced and consistent beneficial impact on plants (Gao et al., 2021). While the potential of endophytes is evident, several research gaps remain: a limited understanding of the specific signalling pathways and molecular mechanisms underlying endophyte-plant interactions, a lack of comprehensive studies on the long-term effects of endophyte application on soil microbial communities and ecosystem health, and a need for efficient and cost-effective methods for endophyte isolation, characterisation, and mass production. These knowledge gaps underscore the need for further research on endophyte-plant interactions, engage the audience in the scientific process, and pique their interest. This review examines how the secondary metabolites of endophyte microbes are the next generation of biopesticides for crop disease management. By exploring the diverse array of secondary metabolites produced by endophytes and their role in suppressing plant diseases, this manuscript aims to inspire the audience about the potential of endophytes as a sustainable and environmentally friendly alternative to synthetic pesticides, thereby paving the way for improved crop health and agricultural practices.

## 2 Secondary metabolites

Microorganisms exhibit prolific biosynthetic capabilities, generating structurally diverse bioactive metabolites with profound implications for human health and agricultural systems. These low-molecular-weight (LMW) molecules, synthesised predominantly during the post-exponential growth phase, function as ecological mediators by enhancing microbial fitness and competitive advantage (Romano et al., 2018). Categorised into primary metabolites ubiquitous across taxa and essential for cellular homeostasis and specialised secondary metabolites, this chemical repertoire encompasses terpenoids, phenolics, and nitrogenous compounds, which serve as foundational scaffolds for pharmaceuticals and agrochemicals (Erb and Kliebenstein, 2020). This research is significant because it provides a comprehensive understanding of microbial biosynthesis, ecological functions, and applications in agriculture and medicine, thereby advancing these fields.

**Figure 1:**
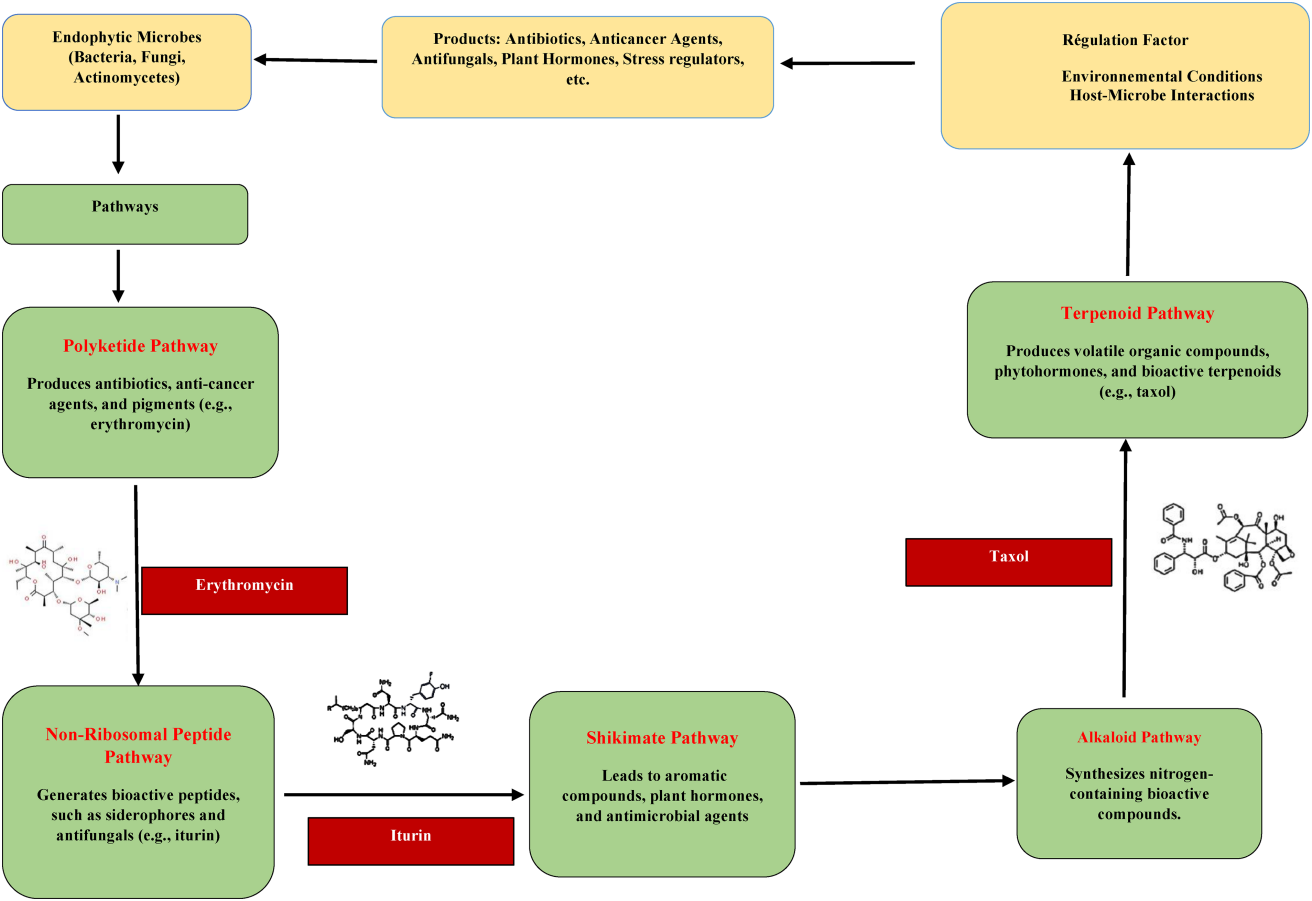
Pathways for the synthesis of some secondary metabolites.

Secondary metabolites of microbial and plant origin demonstrate pleiotropic pharmacological activities, including antineoplastic, anti-inflammatory, antioxidant, and antiplasmodial effects (Bhatti et al., 2022). Ecologically, these compounds antagonise phytopathogens through multifaceted mechanisms, including the biosynthesis of antibiotics (e.g., polyketides, non-ribosomal peptides), phytotoxic alkaloids, enzyme-inhibitory tannins, redox-active phenolics, volatile organic compounds (VOCs), and sulphur-containing antimicrobials (Breen et al., 2015). Endophytic microorganisms, which occupy intracellular and intercellular niches within plant tissues, represent an underexplored reservoir of bioactive entities, often exhibiting host-specific metabolic mimicry (Taritla et al., 2021). Biosynthetic pathways of secondary metabolites originate from primary metabolic intermediates. Acetyl-CoA, a central node in primary metabolism, acts as a precursor for polyketide synthases (PKSs), generating aflatoxins and terpenoid backbones like carotenoids (Zhang et al., 2021; Figure 1). Under biotic and abiotic stress, plants upregulate secondary metabolite production via modulation of phyto-regulatory hormones, gibberellins, auxins (e.g., indole-3-acetic acid), salicylic acid, and cytokinins—which concurrently enhance disease resistance and orchestrate growth-defence trade-offs, endophytic taxa such as *Trichoderma* spp. (Shaffique et al., 2022). Synthesise dual-function metabolites that directly inhibit phytopathogens while inducing systemic resistance via jasmonate/ethylene signalling pathways, thereby contributing to disease-suppressive rhizospheres (Vinale et al., 2014). Co-evolutionary dynamics are evidenced by fungal endophytes producing secondary metabolites iso-functional to host phytochemicals, suggesting horizontal gene transfer or convergent evolution (Nyalo et al., 2023). Microbial metabolites, encompassing immunomodulatory agents, enzyme inhibitors, antitumor agents, and allelochemicals, mediate complex ecological interactions, ranging from symbiosis to antagonism (Jayaprakashvel and Mathivanan, 2011). Microbes’ genomic architecture encodes vast biosynthetic gene clusters (BGCs), offering biotechnological potential for crop protection and yield enhancement. Endophytes enhance nutrient acquisition through siderophore-mediated iron chelation and phosphate solubilisation while modulating phyto-hormonal crosstalk (e.g., auxin-cytokinin balance) to optimise root architecture and nutrient uptake (Mehta et al., 2019). This metabolic synergy highlights the crucial role of microbial chemical ecology in informing and promoting sustainable agriculture and the discovery of natural products.

## 3 Synthesis of secondary metabolite compounds by endophytic microbes

Endophytes are symbionts that live inside the plant for most of their life cycle without harming the host plant. Utilising these natural symbionts provides a chance to increase crop yields while minimising agricultures negative environmental effects (Kandel et al., 2017). Endophytic microorganisms—encompassing bacteria, fungi, and actinomycetes—possess a high biosynthetic capacity, producing structurally diverse secondary metabolites with significant ecological and agronomic relevance (Berendsen et al., 2018). While primary metabolism sustains cellular growth and homeostasis, secondary metabolism operates as a dynamic, adaptive process, producing non-essential compounds that enhance survival in response to environmental challenges. These metabolites are categorised into distinct classes, including alkaloids, terpenoids, flavonoids, lignans, coumarins, xanthones, saponins, quinones, and specialised enzymes, which collectively mediate biotic interactions and stress resilience (Singh and Kumar, 2023; Kamat et al., 2020). Secondary metabolites originate from intermediates of primary metabolic pathways, with acetyl-CoA serving as a pivotal precursor for polyketides (e.g., aflatoxins) and terpenoid derivatives (e.g., carotenoids) via polyketide synthase (PKS) and terpene synthase (TPS) enzymatic cascades (Zhang et al., 2021). Carbon flux regulation ensures metabolic equilibrium: excess carbon generated during primary metabolism is diverted into secondary metabolite synthesis, which can be remobilised to fuel primary processes during nutrient limitation (Thirumurugan et al., 2018). Under abiotic or biotic stress, plants activate stress-responsive pathways that integrate microbial-derived secondary metabolites, triggering phytohormonal signalling networks (e.g., gibberellins, cytokinins, auxins like indole-3-acetic acid, and salicylic acid) to modulate growth-defence trade-offs and physiological adaptation (Shaffique et al., 2022; Nanda et al., 2019). The biocatalytic potential of endophytes is harnessed for sustainable crop protection, offering an alternative to synthetic agrochemicals. Bacterial genera such as *Bacillus*, *Pseudomonas*, *Agrobacterium*, *Burkholderia*, and *Enterobacter*, alongside fungal taxa including *Trichoderma*, *Aspergillus*, *Penicillium*, and *Gliocladium*, and actinomycetes like *Streptomyces* and *Micromonospora*, produce antimicrobial alkaloids, phenolics, terpenoids, and hydrolytic enzymes that directly suppress phytopathogens (Köhl et al., 2019; Table 1). These metabolites function through multifaceted mechanisms, including disrupting pathogen membranes, inhibiting quorum sensing, chelating essential metals, and inducing systemic resistance in host plants. Critically, microbial secondary metabolites contribute to rhizosphere competence and niche colonisation, with non-pathogenic *Fusarium* strains and *Trichoderma* spp. Synthesising metabolites that prime plant immune responses while enhancing nutrient acquisition (e.g., siderophores, phosphate solubilisers; Kamat et al., 2020). This metabolic synergy highlights the evolutionary optimisation of endophyte-plant symbioses, positioning microbial-derived compounds as key components in developing resilient agricultural systems.

**Table 1:** A list of secondary metabolites produced by microbes that have been used in plant disease management in present-day agriculture.

S. no.	Endophytic microbes	Isolated plant	Bioactive compounds	Results	Reference
Endophytic bacteria					
1.	*Bacillus velezensis*	*Chinese catalpa*	Phenol, benzothiazole, pyrazine, 2- tridecanone	Bacterial strain ZSY-1-strain secreted volatile organic compounds that showed strong antifungal efficacy against both *A. solani* with 83.0% inhibition rate and *B. cinerea* 92.1% inhibition rate.	Gao et al. (2017)
2.	*Pseudomonas chlororaphis*		Pyrrolnitrin	Pseudomonas species secrete two distinct antifungal chemicals, pyrrolnitrin and phenazine-1-carboxamide, which inhibit *F. graminearum* mycelial growth.	Huang et al. (2018)
3.	*Burkholderia gladioli*	*Lycoris aurea*	Toxoflavin	Bacteria secrete Toxoflavin effectively inhibited the growth of several fungal infections, including *Candida albicans*, *Aspergillus Fumigatus*, and *Cryptococcus neoformans*.	Li et al. (2019)
4.	*Pseudarthrobacter phenanthrenivorans*	*Pellaea calomelanos*	Auxin, salicylic acid, jasmonic acid	A number of potential genes were found to be involved in transport, adhesion, motility, membrane, proteins, secretion, and delivery systems, and stress protections. MHSD1 gene is essential for nitrogen fixation and siderophore synthesis	Tshishonga and Serepa-Dlamini (2020)
5.	*Bacillus subtilis*	Cereal crop	mycosubtilin	The potential of mycosubstilin as an agent of biological control by efficiently preventing fungal growth by 48 to 49%, causing fungal cell damage, lowering mycotoxin production and changing the expression of genes involved in toxin manufacture.	Yu et al. (2021)
6.	*Bacillus cereus*	*Zanthoxylum bungeanum*	Pyrrole-2-carboxylic acid	The endophytic bacteria produce pyrrole-2-carboxylic acid (PCA), which causes significant membrane disruption in bacterial cells and ha a broad-spectrum antibacterial action against a variety of foodborne pathogens.	Yue et al. (2022)
7.	*Bacillus velezensis*	Tobacco and potato	--	It was observed that the endophytic bacterium *Bacillus velezensis* E9 produced bacillaene, that inhibits *R. solanacearum*.	Li et al., 2023
Endophytic fungi					
8.	*Saprophytic fungi*	Cereal tissue	--	Yeast and species from the *Trichoderma* genus were among the saprophytic fungi that were evaluated for their ability to effectively combat *Fusarium Graminearum* and *F. culmorum* on wheat straw, as well as *Fusarium proliferatum* and *F. verticillioides* on maize stalks. A potent antagonist, *Clonostachys rosea* was found to regularly lower *Fusarium graminearum* and *Fusarium culmorum* aporulation on wheat straw by 85–99% and 91–100%, respectively.	Luongo et al. (2005)
9.	*Trichoderma* spp.*, A spergillus, Penicillium* spp.	*Anethum graveolens*	Ethyl acetate	*Trichoderma* spp. (16.3%), *Penicillium* Spp. (14.6%), *Aspergillus Spp*. (11.6%), have the strongest antibacterial properties.	El-Zehery et al. (2024)
10.	*Trichoderma brevicompactum*	Garlic	--	*Trichodermin* has a 0.25μgml^−1^ Ec50 and significantly inhibits *R. solani*. Additionally, *Trichoderma* has a significant EC50 of 2.02 μgmL^−1^ against *B. cinerea*. The inhibitory action of *Trichoderma* against *C. lindemuthianum*, however, *C. lindemuthianum* had a poor inhibitory effect (EC50 = 25.60μgml^−1^).	Shentu et al. (2014)
11.	*Cochilobolus sativus*	*Artemisia desertorum*	Helminthosporol acid	Use to control antifungal andanti bacterial	Li et al. (2020)
12.	*Trichoderma Brev*	Banana	--	*Trichoderma Brev* T069, a novel biopesticide, was formulated on a substrate made of cassava peels. The biocontrol efficiency of the biological pesticide *T. Brev* T069 against banana fusarium wilt was 64.65%.	Zhang et al. (2021)
13.	*Penicillium Chrysogenum*	*Albizia adianthifolia*	Pyrrolo, dibuty phthalate, hexahydro	The *P. chrysogenum* P03MB2 fractionated extract increased the Anti-HIV activity.	Makhwitine et al. (2023)
14.	*Camptotheca acuminata*		Camptothecin	Endophytic fungus bioactive metabolites provide interesting substitutes for pharmaceutical development, especially considering the increasing threat of antibiotic resistance. These metabolites have demonstrated potential as immunomodulators, antioxidants, and antimicrobial.	Ajadi et al. (2024)
15.	*Neopestalotiopsis* sp.*, Penicillium* sp.*, Aspergillus* sp.	Bolivia	Terpenes, Flavonoids, Phenolics	Ethyl acetate extract from *Penicillium lanosum* and *Penicillium radiatolobatum* demonstrated minimum inhibitory concentration (MICs) that ranged from 31.25 to 500 μg/mL against both gram-positive and gram-negative pathogens, including *B. cereus*, *S.aureus, L. monocytogenes, E. coli* and *S. enterica.* In this investigation, extracts from *Aspergillus* SMB-27 and *Neopestalotiopsis* SMB-23 exhibited no action up to 2000 μg/mL.	Mendieta-Brito et al. (2024)
16.	*Curvularia eragrostis*	*Helecteris isora*	Alkoloids, phenols, polyketides, quinones, steroids, peptides	*C. eragrostis* secret beneficial secondary metabolites that can inhibit the growth of harmful phytopathogens and help in stress management.	Salehi and Safaie (2024)
Endophytic actinomycetes					
17.	*Streptomyces violaceusniger*	--	Polyketides, Beta Lactams, Peptides, Plant hormones	Certain strain of *Streptomyces* spp. Effectively suppress fungal growth by breaking down the chitin in fungal cell walls, which further strengthens their function in disease management. They also stimulate plant growth by secreting growth hormones. *Streptomyces specie*s are important for soil health and in integrated pest management.	Devi et al. (2022)
18.	*Burkholderia*	Soyabean	--	MS455, a strain of *Burkholderia,* showed strong antifungal activity against growing *A. flavus*	Jia et al. (2022)
19.	*Streptomyces lydicus*	--	Oxadixyl, chloreturon, S- metolacholar, fentrazamide	*S. lydicus* extract damaged plasma membranes and caused *A. alternata*’s cytoplasm to extravasate. The antifungal properties of *S. lydicus* extracts were facilitated by the accumulation of ROS.	Wang et al. (2022)
20.	*Streptomyces, Nocardiodes, pseudonocardia*	*Artemisia herba-alba* and *Artemisia judaica*	Cyromazine, Nitrophenol, Diazinon	*Streptomyces* sp. ES2 caused 50% death of the L- larvae after 48 h treatment. Additionally, after 72 h, the treated larvae showed some toxicity effects. And some larvae produced larval-pupal or pupal-larval intermediates because they were unable to pupate from the larval stage to the pupal stage.	Diab et al. (2023)
21.	*Streptomyces, streptoverticillium, actinoplanes*	--	Iturin, siderophores, koningins	Microbial metabolites demonstrate significant potential, encompassing direct pathogen elimination, depriving nutrients (by using siderophores), and inducing oxidative stress. These processes help Microbial metabolites manage plant disease more effectively overall.	Dixit and Kumari (2023)
22.	*Streptomyces* spp.	Grains	--	According to the finding, co-inoculation reduced the biomass of fungi by up to 71% and inhibited DON generation by up to 99%. This demonstrates how these streptomyces strains may be used in farming operations to control Fusarium head blight and lower mycotoxin contamination.	Colombo et al. (2020)
23.	*Bacillus species*	*Thymus vulgaris*	Auxin synthesis, siderophore production, lytic enzyme, and phosphate solubilisation	Different pathogens and bacterial strains have varying percentages of inhibition, The strain of *Enterobacter xiangfangensis* EGY31, *Bacillus sonorensis* Egy11, and *Bacillus sub* sp. *Inaquosorum* EGY 15 showed the highest zone of inhibition against *Alternaria solani* (F3–77%), *F. fulva* (F2–71%), and *Fusarium oxysporum f.* sp. (F1–70%).	Muhammad et al. (2024)
24.	*Pseudomonas* spp.*, Arthrobacter, Bacillus and serratia*	*Solanum Lycopersicum, Psidium guajava, Pinus merkusii, Dendrocalamus asper, Albizia chinensis,* and *Theobroma cacao L,* respectively.	Phenols, flavonoids, tannins, terpenoids, steroids, saponins, and alkaloids	In vitro testing *Meloidogyne* sp. Revealed a high fatality rate of 95.46% for *Bacillus thuringiensis AK08.* Compared to other microorganisms, including *Bacillus cereus* (81.05%) and *Pseudomonas aeruginosa* (82.93%).	Maulidia et al. (2020)

### 3.1 Endophytic bacteria

**Figure 2:**
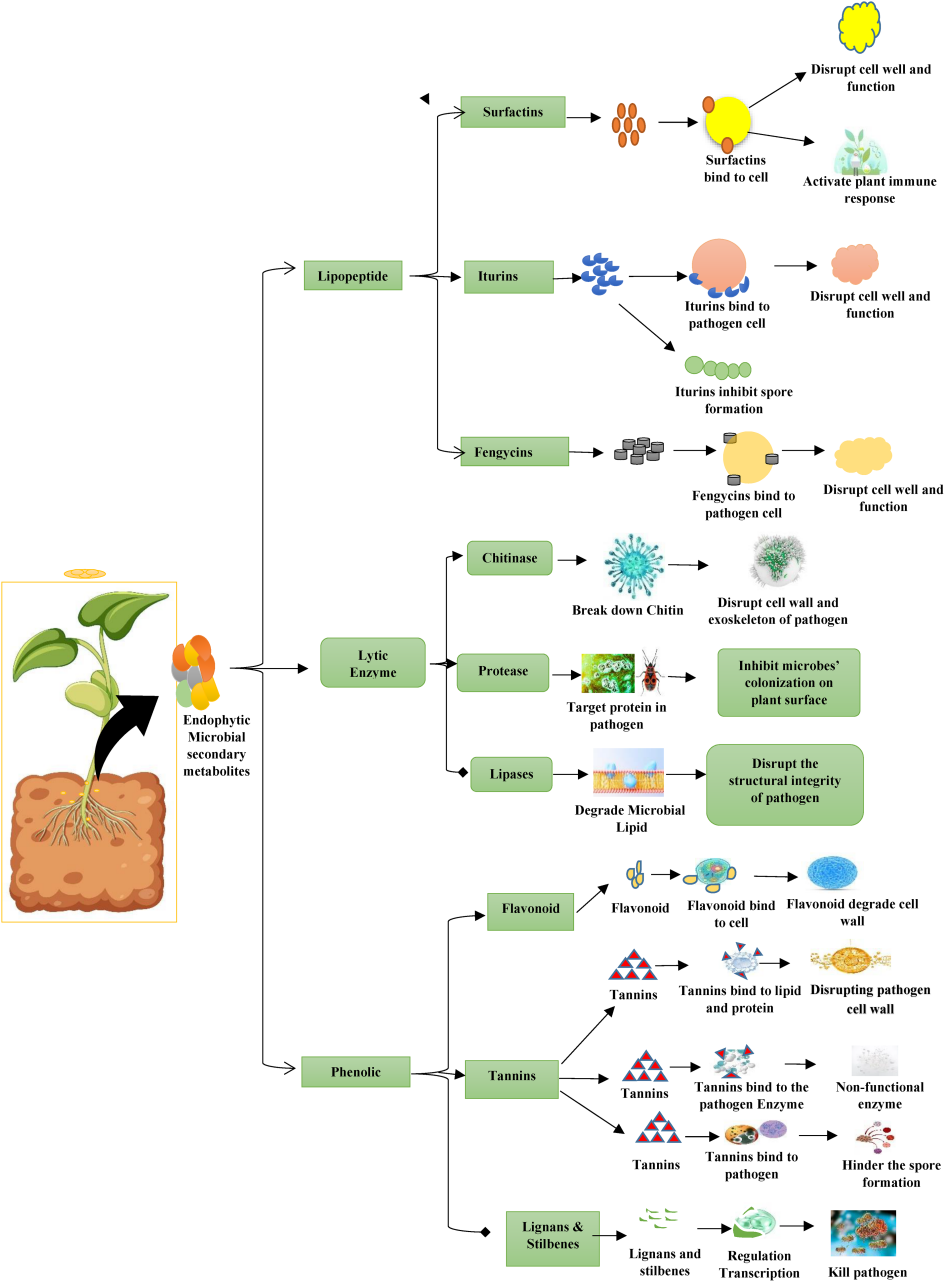
Metabolites of plant associated endophytic microbes.

Bacterial endophytes, primarily belonging to the classes Proteobacteria, Firmicutes, and Actinobacteria, form symbiotic associations with plants, establishing host-specific interactions that are critical for plant health. These microbes, including genera such as *Bacillus*, *Pseudomonas*, *Paenibacillus*, and others, exhibit potent antibiotic activity against phytopathogens while promoting the growth of their host. Their integration into plant microbiomes underscores their role as keystone agents in sustainable agriculture, reducing the need for synthetic agrochemicals and thereby promoting safer, more sustainable farming practices. Central to their biocontrol efficacy are lipopeptides and cyclic amphiphilic metabolites produced by *Bacillus* spp., which include surfactins, iturins, and fengycins. These compounds disrupt pathogen membranes, inhibit quorum sensing, and prime plant immune responses, thereby suppressing diverse phytopathogens (fungi, bacteria, oomycetes) while enhancing ecological fitness and rhizosphere colonisation (Ongena and Jacques, 2008; Kumar and Johri, 2012; Figure 2).

Additionally, *in-vitro* studies frequently report minimum inhibitory concentrations (MICs) or effective concentrations (ECs) for these lipopeptides, setting useful baselines. For instance, iturin A typically exhibits full inhibition of susceptible fungal pathogens at concentrations around 50–100 μg mL^−1^, whereas plipastatin (a fengycin family member) often requires approximately 100 μg mL^−1^ for complete suppression under laboratory conditions (Kumar and Johri, 2012; Yu et al., 2021). However, these *in vitro* thresholds do not necessarily predict efficacy in planta, where complex host and environmental interactions prevail.

In planta experiments reveal effective elicitation windows for disease suppression at significantly lower molar concentrations. Surfactin suppresses bacterial diseases in hydroponic and rhizosphere applications at 0.06–0.5 μM in rice and 1–4 μM in tomato, whereas root-applied iturin A shows activity near 0.5–2 μM in rice with more variable results in tomato hosts. Notably, disease suppression exhibits a non-linear dose–response curve, as concentrations exceeding certain thresholds (for example, above 2 μM) may negate beneficial outcomes, consistent with patterns observed for immune system elicitors (Ongena and Jacques, 2008; Bacelar et al., 2024). These lipopeptides engage plant defence pathways differently: fengycin and surfactin consistently induce transcriptional responses akin to induced systemic resistance (ISR). At the same time, iturin A also displays strong direct antifungal activity alongside context-dependent immune activation. Specific defence genes, for example, PR1, chitinase (CHI), and peroxidase (POD) are differentially upregulated depending on the lipopeptide and infection status (Kumar and Johri, 2012; Bacelar et al., 2024).

Despite promising bioactivities, field application of lipopeptides is hampered by formulation challenges, notably poor adhesion on leaf surfaces and susceptibility to UV- and photo-oxidative degradation, which diminish surface persistence and efficacy. Strategies to overcome these barriers include micro- and nano-encapsulation technologies that protect active compounds, use of stickers and spreaders to improve leaf wetting and retention, and timing applications during dawn or dusk to minimise UV exposure (Le Cocq et al., 2017; Sarma et al., 2021). These formulation advances are essential to maximise the delivery and stability of lipopeptide-based biopesticides in real-world agroecosystems.

Furthermore, Structural variability in fatty acid chains and peptide moieties underpins their functional diversity, enabling targeted antagonism of pathogens such as *Fusarium graminearum*. For instance, mycosubtilin from *B. subtilis* ATCC6633 destabilises hyphal cell walls and plasma membranes, blocks conidial germination, and reduces spore viability in a dose-dependent manner (Yu et al., 2021). Endophytes further engage in cross-kingdom signalling via microbial quorum-sensing molecules (e.g., cyclodipeptides and N-acyl homoserine lactones) that plants recognise to modulate defence pathways and nutrient uptake (Ortiz-Castro and López-Bucio, 2019). Moreover, bacterial synthesis of ACC deaminase mitigates ethylene-mediated stress by cleaving 1-aminocyclopropane-1-carboxylate (ACC) into *α*-ketobutyrate and ammonia, enhancing root colonisation and nitrogen availability (Sun et al., 2009). ACC deaminase is an enzyme produced by certain plant-associated bacteria that breaks down 1-aminocyclopropane-1-carboxylate (ACC), which is the immediate precursor of the plant hormone ethylene. This enzymatic reaction converts ACC into α-ketobutyrate and ammonia, effectively lowering ethylene levels in plants (Glick, 2005). Ethylene is a plant hormone that, at low levels, regulates normal growth processes such as root and seed development but, when overproduced under stress conditions (termed “stress ethylene”), it can trigger negative effects like premature leaf senescence, abscission, and inhibited root elongation (Singh and Singh, 2025). For example, studies showed that endophytes producing ACC deaminase enhance root proliferation and mitigate stress-related growth inhibition by lowering ethylene accumulation (Orozco-Mosqueda et al., 2020). Additionally, this enzymatic activity, coupled with hydrolase production and phytohormone secretion (e.g., auxins, cytokinins), primes plants for drought tolerance and pathogen resistance, illustrating the multifunctional role of endophytes in stress adaptation (like salinity, drought, or pathogen pressure; Singh et al., 2017; Shi et al., 2014). Furthermore, the translational potential of these interactions is evidenced by commercialised biocontrol agents that leverage endophytic metabolites. Bacterial-based formulations such as Cedomon® (*Pseudomonas chlororaphis* MA342) and Mycostop® (*Streptomyces griseoviridis*), as well as fungal products such as Contans® WG (*Coniothyrium minitans*), have demonstrated successful market applications (Lahdenperä et al., 1991; Tombolini et al., 1999). Synergistic inoculation of medicinal plants with endophytes, such as *Bacillus mojavensis*, enhances bioactive compound synthesis, demonstrating dual benefits for crop protection and phytochemical yield (Zhou et al., 2016; Camele et al., 2019). The convergence of microbial secondary metabolites-lipopeptides, enzymes, phytohormones, and host signalling networks positions endophytes as pivotal players in next-generation biopesticide development. Therefore, by leveraging their biosynthetic versatility and plant-microbe communication systems, these microbes offer a sustainable paradigm for crop disease management, reducing agrochemical dependency while enhancing food security.

### 3.2 Endophytic fungi

Endophytic fungi, integral components of plant microbiomes, exhibit unparalleled biosynthetic versatility, producing structurally diverse secondary metabolites directly relevant to sustainable crop protection (Rustamova et al., 2020). These fungi colonise plant tissues without inducing pathogenesis, instead synthesising bioactive compounds, including alkaloids, terpenoids, steroids, flavonoids, anthraquinones, and cyclic peptides, that function as insecticidal agents, antibiotics, and natural antioxidants (Singh et al., 2021). Their ecological and agricultural significance is exemplified by *Piriformospora indica*, a root-colonising basidiomycete that enhances plant growth and stress tolerance through phytohormonal modulation and nutrient uptake optimization (Kumar and Johri, 2012). The biocatalytic potential of endophytic fungi is exemplified by genera such as *Trichoderma*, *Penicillium*, *Aspergillus*, and *Talaromyces*, which dominate commercial biopesticide development due to their prolific metabolite output. *Trichoderma* spp., widely deployed as plant growth-promoting fungi (PGPF) and biological control agents (BCAs), produce volatile and non-volatile antifungal compounds, including peptaibols, gliotoxin, 6-n-pentyl-6H-pyran-2-one (6PP), and harzianopyridone, which disrupt pathogen membranes, inhibit spore germination, and suppress soil-borne diseases (Jalil et al., 2022; Carreras et al., 2020). Similarly, *Aspergillus* species synthesise aflatoxins and mycotoxins, posing global food safety risks, yet endophytic bacteria like *Burkholderia* sp. MS455 counterbalance this threat by producing occidiofungin, a broad-spectrum antifungal agent targeting *Aspergillus flavus* and other pathogens (Makhwitine et al., 2023). The dual role of fungal metabolites as both threats (e.g., aflatoxins) and solutions (Imran et al., 2020) highlights the need for precision in biopesticide development (Mawcha et al., 2025). Emerging strategies leverage endophytic fungi to neutralise mycotoxins while enhancing plant resilience (Fite et al., 2023). For instance, *Phlebiopsis* (Żółciak et al., 2020), and *Gliocladium* species secrete hydrolytic enzymes (Żółciak et al., 2020) and secondary metabolites that degrade fungal cell walls, offering dual-action biocontrol against pathogens like *Fusariu*m and *Rhizoctonia* (Shahid et al., 2021).

Furthermore, fungal-derived anthraquinones and cyclic peptides exhibit insecticidal properties, reducing reliance on synthetic pesticides (Singh et al., 2021). Fungal-based biocontrol products demonstrate progress from lab to field, though scalability and metabolite stability remain challenges. Advances in genomics and metabolomics are unravelling biosynthetic gene clusters (BGCs) in fungi like *Tilletiopsis* and *Laetisaria*, enabling engineered overproduction of target metabolites. Synergistic integration of fungal endophytes with bacterial consortia, as demonstrated by *Burkholderia*-*Trichoderma* co-inoculants, enhances disease suppression and crop yield, aligning with the “Next-Generation Biopesticide” paradigm (Makhwitine et al., 2023). Thus, endophytic fungi represent an underexplored reservoir of bioactive metabolites poised to revolutionise crop disease management. Therefore, harnessing their biosynthetic diversity and ecological adaptability, these organisms offer sustainable alternatives to chemical fungicides that address global food security challenges. Future research must prioritise metabolite stability, field efficacy trials, and regulatory frameworks to accelerate the adoption of fungal-derived biopesticides in agroecosystems.

### 3.3 Endophytic actinomycetes

Endophytic actinomycetes, renowned for their biosynthetic prowess, produce a diverse array of secondary metabolites, including lactones, amides, amines, nucleosides, and macrolides, with potent herbicidal and insecticidal properties, positioning them as keystone agents in sustainable crop disease management (Shi et al., 2014). Historically pivotal in biocontrol since the early 20th century, actinomycetes laid the foundation for antibiotic discovery, with streptomycin, tetracycline, and erythromycin revolutionising agriculture and medicine (Diab et al., 2024). Despite yielding only 20–25% of the ~10,000 bioactive compounds identified from endophytes, actinomycetes remain a critical reservoir for novel biopesticides, particularly in an era of escalating pesticide resistance (Aamir et al., 2020). Endophytic actinomycetes engage in mutualistic relationships with host plants, enhancing growth through phytohormone synthesis and nutrient solubilisation. Their dual role as biofertilizers and biopesticides, offering eco-friendly alternatives to synthetic inputs, underscores their agro-biotechnological potential (Kaari et al., 2023).

Furthermore, the enzymatic capacity of endophytic actinomycetes for pollutant degradation aligns with integrated bioremediation strategies. These strategies involve using multiple biological agents, including actinomycetes, to enhance soil health and crop resilience by breaking down pollutants and improving nutrient availability (Tiwari et al., 2023). The emergence of insecticide-resistant pests necessitates innovative solutions (Diab et al., 2023) demonstrated the efficacy of secondary metabolites from 70 endophytic actinobacteria against *Spodoptera littoralis* with *Streptomyces* sp. ES2 (isolated from *Artemisia herba-alba*) exhibited remarkable larvicidal activity. LC-QTOF-MS–MS profiling revealed ES2’s metabolite suite, enriched in macrolides and peptides, while molecular docking elucidated their binding affinity to insect acetylcholinesterase, disrupting neuronal function (Diab et al., 2023). This multi-omics approach validates actinomycetes as a source of mechanism-specific biopesticides capable of overcoming resistance. Advancements in genomics and metabolomics are unravelling biosynthetic gene clusters (BGCs) in actinomycetes, enabling targeted exploitation of pathways for metabolite overproduction (Dinesh et al., 2017). These BGCs are groups of genes that work together to synthesise, modify, regulate, and transport complex bioactive compounds (Govindhan et al., 2024). Some common BGC classes found in these microbes include non-ribosomal peptide synthetases (NRPSs), type I and II polyketide synthases (PKSs), terpene biosynthesis, and ribosomally synthesised and post-translationally modified peptides (RiPPs). Each of these classes produces compounds that are highly effective against fungi, bacteria, and insects (Omomowo et al., 2023). Recent progress in genome mining, especially with tools such as antiSMASH, has shown that each Streptomyces genome typically harbours dozens of BGCs. These BGCs vary widely across strains, and some are hybrid clusters that combine different biosynthetic pathways (Govindhan et al., 2024). This wide range of metabolite structures and activities, which are important for crop protection applications (Ayilara et al., 2023), is directly related to the rich genetic diversity. For instance, lipopeptides produced by NRPS and macrolides produced by PKS are both effective against fungal pathogens. Lantipeptides, on the other hand, target bacterial threats. This also affects how formulations are made to fit their chemical properties (Mishra et al., 2015). Moreover, case studies emphasise particular *Streptomyces* strains abundant in BGCs that exhibit extensive biocontrol activity, underscoring the significance of BGC richness as an indicator of metabolite diversity and biopesticide potential (Omomowo et al., 2023; Govindhan et al., 2024). Coupled with CRISPR-based engineering, these tools promise tailored biopesticides with enhanced stability and specificity. Field trials integrating actinomycete consortia, such as *Streptomyces-Bacillus* synergies, highlight their potential in integrated pest management (IPM) systems, reducing agrochemical dependency. Endophytic actinomycetes epitomise the convergence of ecological symbiosis and biotechnological innovation, offering a roadmap for next-generation biopesticides. Thus, integrating endophytic bacteria, fungi, and actinomycetes into agricultural systems heralds a transformative era in sustainable crop disease management. Endophytic bacteria, such as *Bacillus* and *Pseudomonas* (Sun et al., 2013), leverage lipopeptides and phytohormones to suppress pathogens, enhance nutrient uptake, and prime systemic resistance, while their fungal counterparts (e.g., *Trichoderma, Aspergillus*) deploy volatile organic compounds, peptaibols, and mycotoxin-neutralising metabolites for dual-action biocontrol and stress resilience (Fouda et al., 2019; Lashin et al., 2021). Endophytic actinomycetes, exemplified by *Streptomyces* spp. (Kämpfer, 2006) offer unparalleled biosynthetic diversity, yielding macrolides and enzyme inhibitors to combat pesticide-resistant pests and improve soil health through bioremediation (Vurukonda et al., 2018). Advances in multi-omics profiling, CRISPR-based engineering, and microbial consortia design are poised to unlock synergistic interactions among these microbes, optimising metabolite stability and field efficacy (Sahoo et al., 2025). Additionally, commercialization of actinomycete-derived biopesticides faces several inherent challenges that complicate their large-scale production and application. These include slow or inconsistent microbial growth rates, a high oxygen demand combined with sensitivity to shear forces, and long fermentation periods lasting 7 to 14 days (Silva et al., 2022). Additionally, the complexity of the media required for growth can impede oxygen transfer and hinder effective control of microbial morphology. Collectively, these factors increase production costs and introduce batch-to-batch variability, making scale-up difficult and expensive (Silva et al., 2022; Torres-Rodriguez et al., 2022).

To overcome these barriers, process optimisation strategies have been employed, including adjusting the carbon-to-nitrogen ratio in the growth media, optimising inoculum size and agitation speed, and fine-tuning fermentation time and temperature. These efforts have resulted in improvements in bioactive compound yields and inhibitory activity by roughly 10–15% in controlled biocontrol studies. More advanced methods, including fed-batch or perfusion fermentation systems, help stabilise dissolved oxygen levels, while controlling microbial pellet morphology facilitates downstream processing and purification. Selection of appropriate solvents during recovery stages further enhances product retention and reduces losses (Silva et al., 2022). Moreover, for the creation of fungal biopesticides, it is important to have methods for extracting and purifying metabolites. Filtration or centrifugation is used to separate cell-free culture broth from mycelial biomass, thereby facilitating phase separation (Rustamova et al., 2020). Ethyl acetate is the most common solvent for extracting mid-polar secondary metabolites from culture broth. Methanol, dichloromethane, hexane, and ethanol are other options that can be chosen based on polarity (Kusari et al., 2012). After equal-volume solvent partitioning, the mixture is dried over anhydrous salts and rotary evaporated at temperatures no higher than 45 °C to keep thermolabile bioactives (Jalil et al., 2022). Additional purification processes include liquid–liquid partitioning with n-hexane to eliminate lipids and n-butanol for polar fraction enrichment, as well as solid-phase extraction (C18, silica) for rapid desalting and concentration (Carreras et al., 2020). For quick metabolic profiling, thin-layer chromatography (TLC) is used with UV or derivatization (Makhwitine et al., 2023). Normal-phase and reverse-phase flash chromatography, as well as preparative HPLC with C18 gradients, are used for large-scale purification. Size-exclusion chromatography can also be used for complex mixtures (Shahid et al., 2021). Bioassay-guided fractionation loops are necessary to maintain potency, with low-temperature handling and the avoidance of reactive reagents until advanced purification stages. Throughout the process, fractions are tested against target pathogens or pests (Imran et al., 2020). For mass and fragmentation data, structural elucidation uses LC–MS/MS; for detailed structural information, it uses 1H, 13C, and 2D NMR spectroscopy; and for metabolite identification, it uses FTIR as a supporting technique. Analytical HPLC and quantitative LC–MS (Singh et al., 2021) show that purity and quantity are correct. Early stability testing under stress conditions of light, heat, and pH helps with formulation strategies and storage needs (Rustamova et al., 2020). Practices that are good for the environment include using less solvent, recycling, and using greener solvents whenever possible. They also include solid-phase microextraction (SPME) and headspace GC–MS for the analysis of volatile organic compounds (VOCs) important for bioactivity (Jalil et al., 2022). Metabolite polarity and stability profiles are associated with downstream formulation options, including wettable powders, oil dispersions, or encapsulations, and photosensitive compounds are identified as UV protection additives (Mawcha et al., 2025).

From a regulatory standpoint, actinomycete-derived agents have a successful track record, notably the avermectins produced by *Streptomyces avermitilis*, which are well-established in agricultural pest control worldwide (Hariprasad, 2016). These products are backed by extensive toxicological and environmental safety data that serve as a model for dossier preparation for new microbial biopesticides. The accumulated experience from scaling up antibiotic fermentations offers valuable lessons for quality assurance, manufacturing controls, and product stability necessary for agricultural applications, highlighting the importance of consistent process control and residue management (Silva et al., 2022; Torres-Rodriguez et al., 2022). Finally, these commercialization challenges and solutions are linked to cutting-edge innovations such as nano- and micro-encapsulation techniques, the use of cost-effective substrates, and delivery systems based on microbial consortia. These developments aim to improve stability, safety, regulatory compliance, and ultimately adoption by farmers, bridging scientific advances with practical use in sustainable agriculture (Silva et al., 2022).

## 4 Mode of entry and establishment of entophytic microbes in the plant

Endophytic microbes—comprising bacteria, fungi, and actinomycetes, establish symbiotic relationships with host plants by colonising internal tissues, including roots, stems, and intercellular spaces, without eliciting pathogenic symptoms (Basit et al., 2021). This colonisation is facilitated by diverse entry mechanisms: passive infiltration through lateral root junctions, enzymatic degradation of plant cell walls (e.g., cellulases and pectinases), or active penetration via stomata (Synek et al., 2021). For instance, *Enterobacter* sp. SA187 exploits root elongation zones for epiphytic growth, followed by endophytic colonisation at lateral root bases. At the same time, *Bacillus cereus* induces systemic resistance (ISR) against *Botrytis cinerea* through jasmonate/ethylene (JA/ET) signalling, upregulating PR1 proteins and WRKY53 transcription factors (Mengistu, 2020). According to Sarwat (2016), WRKY53 is essential for controlling responses to various stressors, including salicylic acid, hydrogen peroxide, ozone, and dehydration, as well as pathogen infection.

**Figure 3:**
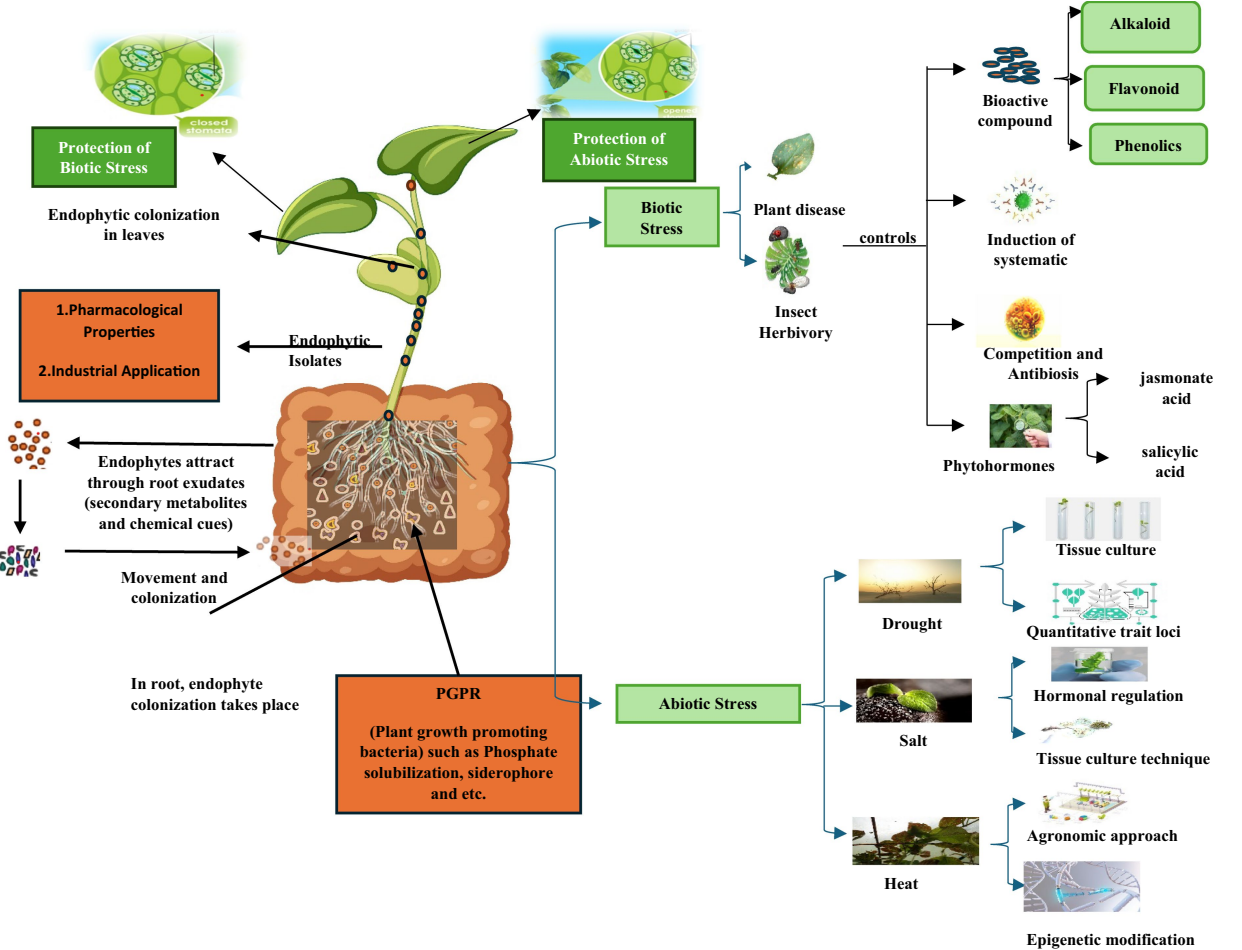
Biotic and abiotic stress management by endophytic microbes.

Drought stress also increases WRKY53 expression (Sun and Yu, 2015). It was demonstrated that WRKY53 not only auto-activates but also controls other WRKY family members (Chen, 2017). Wrky53 normally activates WRKY13, WRKY15, WRKY18, WRKY22, WRKY29, and, WRKY62 while adversely regulating WRKY6 and WRKY42. WRKY 53 generally negatively regulates WRKY6 and WRKY42 while initiating the activation of WRKY13, WRKY15, WRKY18, WRKY22, WRKY29 and WRKY62 (Miao et al., 2004). It was shown that WRKY53 could bind to its own promoter and activate its own expression. WRKY53’s ability to attach to its specific promoter and trigger its own expression. The capacity of WRKY53 to bind to its particular promoter and initiate its own expression was demonstrated (Miao et al., 2013). To determine if HDA9 controls the DNA-binding activity of WRKY53, and create WRKY53-GST. They developed WRKY53-GST to determine whether HDA9 regulates WRKY53’s DNA-binding activity (Zheng et al., 2020). The protein was then employed for gel mobility test using either the mutated version labelled with biotin as a probe or the WRKY53 promoter region with W box (−470 to −380). Researchers developed WRKY53-GST to study whether HDA8 regulates WRKY53’s DNA-binding activity. They create WRKY53-GST to determine whether HDA9 regulates WRKY53’s DNA-binding activity. Both the WRKY53 promoter region with the W box (−470 to −380) and the mutant version labelled with biotin as a probe were used for gel mobility testing. The protein was clearly bound to the wild-type promoter regions but not to the mutant one, and was employed in gel mobility assays with either the WRKY53 promoter region containing the W box (−470 to −380) or a mutant variant of the protein labelled with biotin as a probe. It was evident that the protein was attached to the wild-type promoter site but not to the mutant one. HDA9-GST incubation but not SRT1-GST incubation, SRT1-GST is not incubated with HDA9-GST (Zheng et al., 2020). There is uncertainty regarding HDA9’s capacity to control gene expression cascades involved in plant stress responses and to target specific chromatin sites. By interacting with and controlling the transcription activity, it is possible to eliminate some of the stress response. They found that HDA9 suppresses WRKY53 transcriptional activity by eliminating lysine acetylation sites that post-transcriptionally modify WRKY53. WRKY53, on the other hand, negatively regulates the activity of the HDA9 histone deacetylase. All results indicate mutual antagonistic regulation of each other’s activity by WRKY53 and HDA9. It is still largely unknown how HDA9 targets particular chromatin loci and regulates gene expression networks involved in plant response to stress. Here, we demonstrate that HDA9 represses the stress tolerance response by interacting with and controlling the DNA binding and transcriptional activity of WRKY53, a high-hierarchy positive regulator of stress response. They discovered that WRKY53 is post-translationally modified by lysine acetylation at multiple sites, some of which HDA9 removes. This results in inhibition of WRKY53 transcription activity, while WRKY53 negatively regulates HDA9 histone deacetylase activity. These findings collectively suggest that HDA9 and WRK53 are reciprocal negative regulators of each other’s activities (Zheng et al., 2020). Since 2009, extensive research has been conducted to identify, validate, or fine-map QTLs for FHB resistance in wheat using marker-assisted selection and QTL mapping (Buerstmayr et al., 2020). FHB resistance breeding, including the significance of physical characteristics such as the degree of retained anthers following flowering, their applicability for indirect selection, and the strong correlation between the semi-dwarfing allele Rht-D1b and enhanced anther retention and the severity of FHB. In bread wheat and durum wheat, marker-assisted selection is effective for selecting large-effect QTLs, particularly the most significant resistance QTL, Fhb1 (Steiner et al., 2017; Figure 3). Post-establishment, endophytes orchestrate a molecular dialogue with the host, enhancing stress resilience and pathogen antagonism via secondary metabolites. *Trichoderma arundinaceum*, for example, synthesises trichodiene, a sesquiterpene that primes jasmonate (JA) and salicylic acid (SA) pathways, fortifying defence against necrotrophic fungi (Mengistu, 2020). Similarly, actinomycetes and fungal endophytes secrete bioactive compounds that mitigate abiotic stressors (e.g., drought, salinity) and heavy-metal toxicity, underscoring their dual roles as biopesticides and phytostimulants (Omomowo et al., 2023). Sustainable agriculture’s interdependent objectives during the past 50 years have included preserving the environment and natural resources while limiting the use of synthetic fertilisers and pesticides. These methods promote sustainable agriculture with fewer adverse environmental and climate impacts. As a result, the requirement for utilising appropriate booster techniques to maximise net production has increased. Investigating and using plant-associated beneficial bacteria and their products is the most straightforward approach. Bioinoculants—bioformulations of certain microbial strains on an appropriate carrier—are used to increase crop yields. When utilised as bioinoculants, fungus endophytes offer the host several advantages, including immune response-induced protection against pathogens, mineralisation of vital nutrients, and plant growth promotion (Sharma et al., 2023).

Bacterial endophytes are attracting attention for their contributions to increasing ecological sustainability and agricultural productivity amid the current period of environmental catastrophes and the need for sustainable development. Muhammad et al. play a major role in biodegradation, bioremediation, and nutrient cycling. By secreting low molecular weight organic acid and metal-specific ligands (such as siderophores) that change soil PH and increase binding activity, endophytes have a greater propensity to increase mineral and metal solubility by cells (Anand et al., 2023). Methods, such as the introduction of natural enemies (e.g., fine-mesh nets or natural enemy introductions, such as fine-mesh tricolour), face limitations due to cost, logistical challenges, and inconsistent efficacy (Saucke et al., 2011). In contrast, endophytes offer a sustainable alternative by leveraging host-microbe chemical signalling—mediated by quorum-sensing molecules and phytohormones—to enhance metabolite production and ecological adaptability (Dhayanithy et al., 2019). Quorum sensing is a phenomenon that controls a variety of bacterial traits that may impact plant defence and growth. It is something referred to as bacterial cell–cell communication or bacterial crosstalk. The exudates of roots and bacterial signalling molecules share structural similarities that significantly affect plant health. Numerous bacteria with quorum-sensing systems can be found in the rhizosphere ecosystem, an excellent example of interactions between plants and microbes. The phytochemicals found in plant root exudates, QS signal molecules, and volatile organic compounds generated by microorganisms collaborate to create communications among and across species. Remarkably, their quorum-sensing and quenching capabilities can be directly or indirectly linked to a variety of plant growth-promoting rhizobacterial (PGPR) activities, such as efficient or improved nutrient uptake, root colonisation, nitrogen fixation, antimicrobial compounds, production of plant hormones, and induction of plant defence (Singh and Singh, 2025). This symbiosis-driven metabolic synergy not only suppresses pathogens but also reduces reliance on synthetic agrochemicals, aligning with next-generation biopesticide paradigms.

## 5 Function of metabolites in the interaction between endophytes and host plants

Endophytic microbes predominantly colonise plant roots and rhizospheres, with bacterial taxa such as *Bacillus*, *Pseudomonas*, and *Streptomyces* exhibiting niche-specific localization linked to metabolite exchange and nutrient acquisition (Papik et al., 2020). A dynamic metabolic dialogue governs this symbiotic interaction: endophytes assimilate host-derived nutrients while reciprocally releasing bioactive secondary metabolites, antibiotics, phytohormones, and volatiles—that enhance plant defence, stress tolerance, and growth (Dhayanithy et al., 2019). Phytohormonal crosstalk, particularly involving strigolactones (SLs), is pivotal in sustaining these relationships. SLs, apocarotenoid-derived signalling molecules, regulate hyphal branching in mycoendophytes and prime plant symbiotic pathways by eliciting chitin oligomers, which activate calcium-dependent signalling cascades (Omoarelojie and Van Staden, 2020). Concurrently, arabinogalactan proteins (AGPs), hyperglycosylated hydroxyproline-rich glycoproteins, mediate cell walls interactions critical for microbial adhesion and host recognition during early colonisation (Nguema-Ona et al., 2013). In plant cell walls, arabinogalactans (AGs) are structural polysaccharides. Only a tiny percentage of AGs are linked to pectin and hemicellulose (Held et al., 2015). Moreover, AGs are linked to proteins that form arabinogalactan proteins (AGPs), which are either connected to the plasma membrane by a glycosylphosphatidylinositol (GPI) anchor or present in the plant cell wall with pectin (Silva et al., 2020) Beneficial and pathogenic fungi and bacteria can produce the enzyme required for the full depolymerization of AGs as part of the plant cell wall degradation machinery. The enzymes include endo-*β*-(1,3), β–(1,4) galactanase, β-(1,3/1,6) galactanases, and β-(1,6) galactanases, β- L-arabinofuranosidases, *α*-L-arabinopyranosidases and β-D-glucuronidases. These hydrolytic enzymes may affect how AGPs work and are released during plant-pathogen interactions. It has been suggested that AGPs may be able to stop infections caused by harmful microbes because the breakdown products they produce from pathogens’ hydrolytic enzymes act as damage-associated molecular patterns (DAMPs), triggering the plant’s defence response (Villa-Rivera et al., 2021). Calcium signalling processes that occur after rhizobial and fungal signals are recognised at the root cell plasma membrane, with an emphasis on systemic pathway outputs that are peculiar to mycorrhizal cells. Arbuscular mycorrhizal (AM) symbiosis needs a complex chemical interaction between the fungal partner and the plant host. The rhizosphere receives strigolactones from plant roots, which promote hyphal branching and the germination of fungal spores. AM fungi (AMF) reciprocate by sending signalling molecules known as lipochitooligosaccharides (LCOs) and chitooligosaccharides (COs), which are detected at the plasma membrane of plants (Luginbuehl and Oldroyd, 2016).

The functional synergy between endophytes and plants is exemplified in biocontrol applications. Microbial formulations leveraging *Burkholderia*, *Enterobacter*, and *Streptomyces* suppress pathogens like *Fusarium graminearum*, the causative agent of *Fusarium* head blight (FHB), through competitive niche exclusion and metabolite-driven antagonism (Rojas et al., 2020; Ali et al., 2024). For instance, endophytic fungi asymptomatically colonising wheat spikes secrete antifungal terpenoids and polyketides, outcompeting pathogenic *Fusarium* strains while inducing systemic resistance via jasmonate signalling (Rho et al., 2018). Since 2009, much research has been done to find, validate, or fine-map resistant QTLs in wheat using market-assisted selection and QTL mapping for FHB resistance (Buerstmayr et al., 2020). FHB resistance breeding, including the significance of physical characteristics such as the degree of retained anthers following flowering, their applicability for indirect selection, and the strong correlation between the semi-dwarfing allele Rht-D1b and enhanced anther retention and the severity of FHB. In bread wheat and durum wheat, marker-assisted selection is effective in selecting for large-effect QTLs, particularly the most significant resistance QTL Fhb1 (Steiner et al., 2017). Such interactions underscore the dual role of metabolites as both direct antimicrobial agents and indirect immune primers. The metabolic interplay between endophytes and hosts offers a blueprint for next-generation biopesticides. Colonisation efficiency and metabolite delivery can be optimised by engineering microbial consortia to overexpress SLs or AGP-binding adhesins. Furthermore, integrating multi-omics approaches (metabolomics, transcriptomics) will unravel conserved biosynthetic pathways across taxa, enabling targeted exploitation of novel compounds. Field trials validating microbial formulations against FHB and analogous diseases must prioritise metabolite stability and host-specific compatibility to bridge lab-to-field disparities.

## 6 Studies on the secondary induction role of endophytes in interactions with hosts

Volatile organic compounds (VOCs), a class of microbially derived secondary metabolites, serve as critical mediators of plant-microbe communication, priming systemic resistance against pathogens and herbivores while facilitating cross-kingdom signalling during biotic stress (Poveda, 2021). These compounds, synthesised by bacterial and fungal endophytes such as *Bacillus* spp. and *Trichoderma* spp., underpin their role as biocontrol agents in sustainable agriculture, reducing reliance on synthetic pesticides (Beyari and Alshammari, 2025; Kamali et al., 2022). Examined how tomato (*Solanum lycopersicum*) responded to two functional guilds of nematodes: entomopathogens (Steinernema feltiae below ground, Heterorhabditis bacteriophora and *S. carpocapsae*) and plant parasites (Meloidogyne javanica), as well as an above-ground leaf-mining insect (*Tuta absoluta*). According to the findings, entomopathogenic nematodes (EPNs) decreased the infection of root knot nematodes (RKN) below ground, decreased the preference and performance of herbivores (*T. absoluta*) as hosts above ground, and overlapped plant defence responses by quickly triggering the activity of guaiacol peroxidase and polyphenol oxidase in roots while simultaneously inhibiting this activity in tissues above ground. At the same time, they used transcriptomic analysis to investigate potential plant signalling pathways underlying these interactions. They observed that plant parasites and entomopathogens both induced immune responses in plant roots by expressing the same genes (Wondafrash et al., 2013). The transcripts of secondary metabolites produced in response to the two nematode functional guilds showed a similar regulatory profile and frequently overlapped (Desmedt et al., 2022). Similarly, they demonstrate that EPN decrease the activity of antioxidant enzymes, which, in turn, modulates plant defence against RKN invasion (Khanna et al., 2021). The coevolutionary adaptations, defence-related phytoalexins, while hosts select for microbial strains that optimise. Endophytes modulate host phenylpropanoid and terpenoid pathways, enhancing production of defence-related phytoalexins, while hosts select for microbial strains that optimise. Endophytes modulate host phenylpropanoid and terpenoid pathways, thereby enhancing the production of defence-related phytoalexins. Meanwhile, hosts select for microbial strains that optimise fitness under stress (Mei et al., 2019). Endophytic fungi face dual challenges—breaching structural defences (e.g., cuticles, cell walls, phytoanticipins) and evading immune recognition via plant pattern recognition receptors (PRRs). Successful colonisation hinges on secreted effector proteins, such as cerato-platanins, which act as microbe-associated molecular patterns (MAMPs) to suppress host defences and reprogram secondary metabolite synthesis (Kemen et al., 2015). For instance, fungal endophytes in *Fusarium*-wheat systems secrete chitinases that degrade pathogen cell walls while eliciting host salicylic acid (SA) pathways, demonstrating dual antagonism and immune priming (Torres-Rodriguez et al., 2022). Beyond defence, endophytes contribute to host nutrition through niche-specific mutualistic relationships. Nitrogen-fixing endophytes (e.g., *Bradyrhizobium* in legumes, *Herbaspirillum* in cereals) convert atmospheric N₂ to ammonium, enhancing nitrogen-use efficiency and biomass accumulation under nutrient-limited conditions (Boddey and Dobereiner, 1995). Transmission strategies further shape these interactions: vertical transmission via seeds ensures the intergenerational transfer of symbionts, while horizontal acquisition from soil microbiomes enables the adaptive recruitment of beneficial strains, such as *Pseudomonas* spp. with drought tolerance traits (Luo et al., 2019; Zeng et al., 2023). The integration of VOC-emitting endophytes into crop systems offers a sustainable strategy to enhance resilience against climate-driven stressors. Advances in synthetic biology could engineer microbial consortia to overexpress effector proteins or nitrogenase enzymes, thereby optimising colonisation and metabolite delivery. Field studies must prioritise elucidating transmission efficiency and metabolite stability across agroecosystems to ensure the translational success of endophyte-based bio-pesticides.

## 7 Endophyte microbes promote biotic stress management

Biotic stressors, pathogens, pests, and weeds jeopardise global food security, causing ~40% yield losses in staple crops through direct damage (14% from insects, 13% from pathogens, and 13% from weeds) and compromised physiological resilience (Ferry and Gatehouse, 2010; Dixit and Kumari, 2023). Conventional reliance on synthetic agrochemicals exacerbates environmental degradation, non-target toxicity, and antimicrobial resistance, necessitating urgent adoption of sustainable alternatives (Ngegba et al., 2022; Serrão et al., 2022). Endophytic microbes, symbionts residing asymptomatically within plant tissues, emerge as keystone biopesticides by priming systemic defences, secreting pathogen-antagonistic metabolites, and enhancing nutrient use efficiency (Lacava and Azevedo, 2013; El-Saadony et al., 2022; Figure 3). Endophytes mitigate biotic stress through multipronged strategies:

- Production of ROS, antibiotics (e.g., surfactins, iturins), and hydrolytic enzymes (chitinases, glucanases) disrupts pathogen membranes and quorum sensing (Lata et al., 2018; Minchev et al., 2021).
- Microbial volatile organic compounds (VOCs) and lipopeptides activate salicylic acid (SA) and jasmonate (JA) pathways, upregulating PR proteins and phytoalexins (Bacelar et al., 2024).
- Competitive colonisation of root epidermis and xylem vessels suppresses pathogens like *Fusarium graminearum*, the causative agent of Fusarium head blight (FHB), through spatial and metabolic dominance (Rojas et al., 2018).

For instance, *Trichoderma*-seed coatings enhance germination and seedling vigour in cereals by secreting 6-pentyl-*α*-pyrone, a volatile ketone that antagonises *Fusarium* hyphae while inducing host *β*-1,3-glucanase synthesis (Minchev et al., 2021). Similarly, *Bacillus* spp. Biofertilizers reduce aphid infestations in legumes through plant-mediated JA signalling, demonstrating cross-kingdom potentiation of defence (Johnston-Monje et al., 2016). Biofertilizers that leverage *Pseudomonas*, *Rhizobium*, and *Penicillium* enhance nitrogen fixation, phosphate solubilisation, and micronutrient uptake while concurrently suppressing soil-borne pathogens (Du et al., 2023). Field trials demonstrate that *Pseudomonas chlororaphis* MA342 (Cedomon®) reduces FHB incidence by 60–70% in barley, outperforming synthetic fungicides in drought-stressed conditions (Rojas et al., 2020). However, scalability requires optimising microbial consortia for host specificity, metabolite stability, and compatibility with the soil microbiome. Optimising microbial consortia for host specificity, metabolite stability, and compatibility with the soil microbiome is also necessary. Despite its promise, the interplay between biotic and abiotic stresses (e.g., drought exacerbating pathogen susceptibility) complicates the efficacy of biocontrol. Metabolomics-driven approaches can decode stress-specific metabolite signatures, enabling the development of tailored microbial formulations (Salam, 2023). Advances in CRISPR-based microbiome engineering and in the nano-encapsulation of metabolites (e.g., phenazines, cyclic lipopeptides) promise enhanced delivery and persistence in rhizospheres. Prioritising regulatory frameworks and farmer education will accelerate adoption, aligning with UN Sustainable Development Goals (SDGs) for agro-ecological resilience.

## 8 Secondary metabolites produced by microbes are more convenient than those produced by plants

While plants synthesise secondary metabolites (SMs) such as flavonoids, alkaloids, and tannins to combat biotic stressors and mediate ecological interactions, their production is constrained by low yields (<1% dry weight), developmental stage dependency, and environmental variability (Caldentey and Inzé, 2004; Verma and Shukla, 2015). These compounds, critical for defence against herbivores and pathogens, are energetically costly and often insufficient under acute stress, necessitating the development of alternatives for scalable biopesticide production. Endophytic microbes, in contrast, produce structurally analogous or novel metabolites with enhanced biosynthetic efficiency, overcoming the limitations of plant-derived (Shankar Naik et al., 2019). For instance, endophytic fungi (*Taxomyces*, *Fusarium*) synthesise paclitaxel and camptothecin—chemotherapeutics identical to host plant metabolites, but with higher titers in controlled fermentations, bypassing seasonal and geographical constraints of plant harvests (Venugopalan and Srivastava, 2015).

Advantages of Microbial Biosynthesis Over Plant-Based Systems

- Endophytes generate bioactive molecules, including antimicrobial peptides, terpenoids, and alkaloids, many of which exhibit dual pesticidal and growth-promoting activities (Hashem et al., 2023).
- Unlike plants, microbial SM synthesis is decoupled from environmental variables (light, soil salinity), enabling consistent yields in bioreactors (Ncube and Van Staden, 2015).
- Endophytes like *Trichoderma* and *Penicillium* employ horizontal gene transfer to acquire host-derived biosynthetic pathways, producing hybrid metabolites (e.g., vincristine analogues) with enhanced bioactivity (Zhang et al., 2021).

Despite these advantages, scaling microbial biosynthesis faces significant hurdles. Axenic cultures of endophytes often lose their capacity to produce SM due to epigenetic silencing of biosynthetic gene clusters (BGCs), complicating large-scale fermentation efforts (Filho, 2019). Additionally, gaps in understanding the regulatory networks—such as quorum sensing and epigenetic modifiers—that govern SM synthesis hinder pathway optimisation (Wink, 2016). Economic barriers also persist: high-value metabolites, such as podophyllotoxin, require costly purification processes, while low-value agrochemicals demand cost-effective bulk production methods to compete with synthetic alternatives (Venugopalan and Srivastava, 2015).

However, Advances in synthetic biology and multi-omics are overcoming these limitations. CRISPR-dCas9 systems and small-molecule elicitors, such as histone deacetylase inhibitors, reactivate silent fungal BGCs, unlocking cryptic metabolites with untapped biocontrol potential (Brakhage, 2013). Heterologous expression of endophyte-derived BGCs in tractable hosts like *Aspergillus nidulans* and *Saccharomyces cerevisiae* enables scalable production of plant-toxic compounds (e.g., rotenone analogues) without reliance on plant cultivation (Shankar Naik et al., 2019). Nano-encapsulating volatile metabolites, such as *Trichoderma*-derived 6-pentyl-*α*-pyrone, thereby improving field stability and targeted delivery against soil-borne pathogens (Minchdiscoveries and real-world applications). Thus, microbial secondary metabolites represent a paradigm shift in bio-pesticide development, offering unparalleled structural diversity, biosynthetic scalability, and environmental compatibility compared to plant-derived counterparts.

## 9 Strategies for sustainable and enhanced production of secondary metabolites in endophytes

Endophytic microbes play a critical role in sustainable agriculture, utilising their metabolic versatility to enhance plant nutrient acquisition, stress resilience, and pathogen resistance by targeting the biosynthesis of secondary metabolites (SMs). These microorganisms employ a triad of core strategies to fortify plant health: (1) Nutrient Synergy, via synthesis of auxins, siderophores, and phosphate-solubilising compounds that optimise iron uptake and root architecture; (2) Direct Antagonism, through antimicrobial metabolites (e.g., polyketides, non-ribosomal peptides) that disrupt pathogen membranes and quorum sensing; and (3) Induced Systemic Resistance (ISR), priming plant defences via jasmonate (JA) and salicylic acid (SA) pathways to upregulate pathogenesis-related (PR) proteins and WRKY transcription factors (Velmourougane et al., 2019; Irizarry and White, 2017). For example, *Pseudomonas* spp. secrete pyoverdines, iron-chelating siderophores that starve phyto-pathogens while enhancing host iron bioavailability, exemplifying nutrient-mediated biocontrol (Van Bergeijk et al., 2020).

Building on these foundational mechanisms, researchers harness biotic and abiotic elicitors to amplify SM yields. Microbial co-culture systems, such as *Bacillus-Trichoderma* consortia, stimulate the synthesis of competitive metabolites, enhancing antifungal terpenoids and lipopeptides through interspecies signalling (Backer et al., 2018). Stress mimicry, exposure to sublethal H < sub > 2</sub > O < sub > 2</sub > or heavy metals—activates oxidative stress pathways, boosting antioxidant phenolics and alkaloids (Verma et al., 2018). Complementarily, phytohormone priming with methyl jasmonate (MeJA) or ethylene precursors upregulates biosynthetic gene clusters (BGCs), thereby elevating yields of paclitaxel and vincristine analogues by 3–5-fold (Yang et al., 2022). These approaches exploit endophytic metabolic plasticity to optimise output under controlled conditions. Advances in multi-omics technologies bridge the gap between metabolic potential and practical application. CRISPR-Cas9-mediated promoter knock-in reactivates silent BGCs in *Streptomyces* spp., thereby restoring cryptic antibiotics such as candicidin (Borah et al., 2024). Heterologous expression of BGCs from slow-growing endophytes (e.g., *Taxomyces andreanae*) in industrial hosts (*Aspergillus nidulans*) enables scalable taxol production, circumventing plant-dependent extraction (Kusari et al., 2014). Nano-elicitation—functionalizing chitosan nanoparticles with fungal cell wall components (chitin, *β*-glucans)—mimics pathogen attack, driving *Trichoderma* to overexpress peptaibols and gliotoxin (Minchev et al., 2021). These innovations merge genetic precision with biotechnological scalability, thereby realising their full potential and enhancing metabolite persistence, thereby prolonging pathogen suppression (Sarma et al., 2021). Seed coatings encapsulating *Trichoderma* spp. Induce early ISR, shielding seedlings from *Fusarium* infections while promoting root hair development (Verma et al., 2018). Metagenomic-guided recruitment of native endophytes from disease-suppressive soils revitalises metabolite-driven phyto-immunity in degraded agroecosystems, leveraging natural microbial networks for resilience (Müller et al., 2016).

In a nutshell, integrating elicitation, omics, and ecological strategies forms a holistic framework for harnessing endophytes. By aligning microbial biosynthetic prowess with crop-specific needs, these approaches reduce agrochemical dependency while stabilising yields. Future priorities include field validation of engineered strains, cost-effective nano-formulations, and policy frameworks to accelerate adoption. Cementing endophytes as pillars of sustainable agriculture necessitates interdisciplinary collaboration, transforming lab breakthroughs into farmer-centric solutions to safeguard global food security against escalating biotic and abiotic threats.

## 10 Application of omics to elucidate the interactions between endophytes and hosts

The advent of multi-omics technologies spanning genomics, transcriptomics, proteomics, metabolomics, and metagenomics has transformed our capacity to dissect the molecular dynamics of endophyte-host symbioses, offering unprecedented insights into microbial metabolite biosynthesis, mutualistic signalling, and ecological adaptation. Genomics and metagenomics have unveiled the genetic architecture of endophytes, identifying biosynthetic gene clusters (BGCs) responsible for antimicrobial agents such as lipases, chitinases, and proteases, which disrupt fungal cell walls and suppress pathogenic interactions (Vurukonda et al., 2018). For instance, genome sequencing of the basidiomycete *Ganoderma lucidum* has elucidated terpenoid and polyketide BGCs, establishing it as a model system for studying secondary metabolite regulation (Chen et al., 2012). Complementing this, transcriptomics, particularly dual RNA-sequencing, captures synchronised gene expression in both host and symbiont during colonisation, revealing pathways critical to mutualism, such as nitrogen fixation, phytohormone synthesis (e.g., indole-3-acetic acid, gibberellins), and stress tolerance (Firrincieli et al., 2015; Camilios-Neto et al., 2014).

Proteomics further bridges genotype to phenotype by mapping extracellular and intracellular proteins, including antifungal enzymes, such as proteases, which dominate microbial defence arsenals. Metabolomics, coupled with next-generation sequencing (NGS), profiles secondary metabolites such as siderophores and antibiotics under biotic stress while resolving metabolic pathways activated by environmental inducers (Wang et al., 2016; Jain et al., 2020). These integrative approaches are pivotal for industrial applications, where heterologous expression of BGCs in tractable hosts (e.g., *Streptomyces* spp.) overcomes the low yields of natural strains under laboratory conditions, enabling scalable production of agrochemicals, antifungals, and antitumor agents (Pereira et al., 2024; Khan et al., 2023). *Streptomyces*, a genus renowned for its metabolic versatility, exemplifies the intersection of ecological resilience and biotechnological utility. Thriving in pH-variable environments (6.5 ± 9) and surviving nutrient deprivation via sporulation, its genome encodes stress regulators and diverse carbon/nitrogen metabolic networks, facilitating niche adaptation (Pacios-Michelena et al., 2021). These traits underscore its industrial prominence in antibiotic discovery and sustainable agriculture. Furthermore, evolutionarily, the origins of endophytes remain a subject of contention: endogenous theories posit that ancestral symbionts coevolved with host organelles (e.g., mitochondria, chloroplasts), while exogenous models emphasise horizontal colonisation via root wounds or induced channels (Hondelmann et al., 2020). Multi-omics, with its ability to comprehensively analyse the genetic, transcriptomic, proteomic, and metabolomic profiles of endophytes and their hosts, plays a crucial role in resolving these dynamics. It identifies conserved genes in endophytic lineages and host-specific adaptations that drive mutualistic outcomes, decoding these interactions, omics advances fundamental knowledge and fuels innovations in biocontrol, biofertilizers, and bioremediation, positioning endophytes as keystones of next-generation agricultural and biotechnological solutions.

## 11 Research outlooks of next generation bio-pesticide based on secondary metabolites

Next-generation biopesticides produced from microbial secondary metabolites offer a new avenue for sustainable, chemical-free crop protection. Polyketides, non-ribosomal peptides, and siderophores are some of these substances. They are very good at killing fungi, bacteria, and insects, and they also play important functions in ecology, such as in resource competition, quorum sensing, and communication between hosts and microbes (Govindhan et al., 2024). They are produced mostly during periods of stalled growth or stress, and they are important for protecting plants and maintaining the balance of soil ecology. Modern biopesticides use metabolites from plant-associated and endophytic bacteria to accomplish more than just killing pathogens. They also aim to improve soil health, accelerate nutrient cycling, and increase plant resistance to pests (Ayilara et al., 2023). However, using these metabolites at large scale is very difficult because they are expensive to produce and do not perform well under changing field conditions (Mishra et al., 2015). These problems are being addressed through improvements in fermentation and formulation technologies. Using inexpensive substrates such as cellulose, molasses, and amino acids under optimised nutrient regimes has made microbes more stable and increased the amount of metabolites they produce. Microencapsulation with biopolymers (like alginate and starch), polymeric coatings, oil dispersions, wettable powders, and nano-enabled systems makes sure that the release is controlled, that sensitive compounds are protected from UV damage, and that the product lasts longer and works better in the field (Le Cocq et al., 2017; Sarma et al., 2021; Silva et al., 2022). Formulations for living endophytic bacteria emphasise maintaining life and colonisation potential, whereas metabolite-only solutions emphasise chemical stability and predictable release behaviour. Synthetic biology, CRISPR-based genome editing, and the heterologous production of biosynthetic gene clusters (BGCs) in manageable hosts such as *Pseudomonas putida* and *Bacillus subtilis* enhance emerging research. These methods enable the production of large amounts of high-value metabolites, such as surfactin and iturin, without the metabolic limitations of wild strains (Cavello et al., 2015). The combination of omics-driven discovery platforms (genomics, metabolomics, and metagenomics) has identified BGCs previously hidden in endophytes. These BGCs include new substances that could be used for biocontrol (Omomowo et al., 2023). Formulation readiness gates are increasingly built into development pathways to turn lab discoveries into useful field products. These pre-screening steps assess whether microbes and metabolites can be encapsulated together, ensuring bioactivity is maintained during storage and use. Regulatory frameworks also require strict stability testing, including accelerated ageing, UV exposure, and temperature cycling, under Good Laboratory Practice-like standards to ensure safety and performance remain consistent (Silva et al., 2022; Mishra et al., 2015).

For microbial biopesticides to be long-lasting and useful on a large scale, they need to combine microbial ecology, metabolic engineering, and modern formulation science. Creating synthetic microbial consortia that mimic natural microbial communities can help plants grow and collaborate to produce more metabolites. Commercialisation will accelerate even faster if extraction, fermentation, and cost-effective production lines improve. These diverse tactics work together to demonstrate that endophytic secondary metabolites are important for soil fertility, disease control, and ensuring enough food for everyone in the world. This is the first step towards a more resilient and ecologically friendly agricultural future.

## 12 Conclusion

Endophytic microorganisms and their secondary metabolites represent a promising advancement in sustainable agricultural disease management. Metabolites, including alkaloids, terpenoids, phenofor or an alternative to synthetic pesticides. They directly inhibit infections, stimulate plant systemic resistance, and improve nutrient absorption and stress resilience. Bacterial endophytes, including *Bacillus* and *Pseudomonas,* generate lipopeptides that compromise pathogen membranes and enhance plant immunity, whereas fungal endophytes, such as *Trichoderma* and *Penicillium*, produce antifungal and insecticidal agents. The ecological functions and symbiotic chemical interactions between endophytes and host plants enhance plant growth and defence via molecular processes such as phytohormonal signalling and quorum sensing.

Notwithstanding their potential, substantial research deficiencies necessitate our essential. Understanding the lasting effects on ecosystems, molecular mechanisms, and scalable production techniques is crucial. Incorporating endophytic secondary metabolites into agricultural protection techniques might diminish dependence on chemical pesticides, hence enhancing environmental sustainability and food security. Further study and development are essential to fully exploit the biotechnological potential of endophytes as environmentally benign biopesticides. This domain is primed for investigation and innovation, which is crucial for establishing resilient agricultural systems globally. Endophytes offer a sustainable approach to crop disease management by leveraging their biochemical diversity and plant-microbe communication systems, thereby enhancing food security and reducing reliance on agrochemicals.
